# Post-traumatic vancomycin-resistant enterococcal endophthalmitis

**DOI:** 10.1186/1869-5760-3-42

**Published:** 2013-03-18

**Authors:** Roxane J Hillier, Parnian Arjmand, Gabriel Rebick, Mario Ostrowski, Rajeev H Muni

**Affiliations:** 1 Ophthalmology Department, St Michael's Hospital, 801-61 Queen Street East, Toronto, ON, M5C 2T2, Canada; 2 McMaster University, Hamilton, ON, L8S 4L8, Canada; 3 Infectious Disease Service, St. Michael's Hospital, Toronto, ON, M5B 1W8, Canada

**Keywords:** Endophthalmitis, Enterococcus, Vancomycin resistance, Ocular trauma

## Abstract

The emergence of antibiotic-resistant organisms among severe ocular infections is of grave concern. We describe the first reported case of vancomycin-resistant enterococcal endophthalmitis following ocular trauma, uniquely caused by *Enterococcus gallinarum*. The organism demonstrated intrinsic resistance to ceftazidime and vancomycin but responded favorably to a combination of intravitreal and intravenous ampicillin, plus intravitreal amikacin. When faced with a multidrug-resistant organism, the ophthalmologist must consider alternative antibiotic strategies.

## Dear Editor

The emergence of vancomycin-resistant enterococcal (VRE) endophthalmitis is of grave concern. To date, five cases of VRE endophthalmitis have been reported in the literature, associated with previous ocular surgery or immunosuppression [1-5]. In these cases, the causative organisms were *Enterococcus faecium* and *Enterococcus faecalis.* We describe the first reported case of VRE endophthalmitis following ocular trauma, uniquely caused by *Enterococcus gallinarum.*

## Case description

A 38-year-old male sustained trauma to the left eye while hammering farm machinery without protective eyewear. At presentation, 15 h later, the visual acuity (VA) was hand movements. Anterior segment examination confirmed a 3-mm linear corneal laceration with iris tissue and lens capsule to the wound. A fibrinous reaction and 0.5-mm hypopyon were evident. Fundoscopy revealed a healthy posterior pole, with no vitritis. A CT scan of the orbit identified the presence of a metallic intraocular foreign body (IOFB). The patient proceeded to primary repair, pars plana lensectomy, vitrectomy, removal of IOFB, and partial air-fluid exchange. The patient was left aphakic. Intraoperatively, dense vitritis and florid vasculitis, obscuring the optic disc and vessels, were noted to have developed in the short interval between presentation and surgery. A vitreous specimen was sent for microbiologic evaluation, and intravitreal vancomycin 1 mg/0.1 ml, ceftazidime 2.25 mg/0.1 ml, dexamethasone 0.4 mg/0.1 ml, and intravenous moxifloxacin 400 mg were administered. On post-operative day 1, VA dropped to light perception, with a 2-mm hypopyon and absent red reflex. Initial gram stain of the vitreous specimen identified gram-positive cocci in pairs. A second intravitreal injection of vancomycin 1 mg/0.1 ml was administered empirically. Oral moxifloxacin 400 mg once daily and topical fortified vancomycin, fortified ceftazidime, and prednisolone 1% were commenced. On post-operative day 2, there was no clinical improvement. The primary organism was identified as vancomycin-resistant *E. gallinarum*, sensitive to ampicillin and gentamicin. Light growth of a gram-negative bacillus was also seen. Intravitreal ampicillin 5 mg/0.1 ml and amikacin 0.4 mg/0.1 ml were administered. A 2-week course of intravenous ampicillin 2 g every 4 h was commenced, plus intravenous clindamycin 600 mg every 8 h and oral moxifloxacin 400 mg once daily to cover the gram-negative organism (subsequently identified as an *Acinetobacter* species). Topical therapy was switched to fortified gentamicin and prednisolone 1%. The infection responded quickly to this regime, and topical therapy was tapered over subsequent weeks. Recovery was complicated by subsequent inferior retinal detachment, which underwent successful surgical management. At 7 months post-injury, the VA improves to 20/80 (aphakic correction).

## Comment

Current consensus regarding initial empirical treatment of post-traumatic endophthalmitis is the administration of intravitreal vancomycin and ceftazidime, with adjunctive intravenous vancomycin and ceftazidime, or oral moxifloxacin while awaiting culture results [6]. However, when faced with multidrug resistance or polymicrobial infection, the ophthalmologist must consider alternative antibiotic strategies. Enterococci are gram-positive organisms with intrinsic resistance to many antibiotics including cephalosporins. While frequently sensitive to vancomycin, the clinician must consider VRE particularly in previously hospitalized patients and in areas of high VRE prevalence [3]. Furthermore, certain species including *E. gallinarum* demonstrate intrinsic vancomycin resistance. Intravitreal ampicillin [2,3] and amikacin [2] have been used to successfully treat VRE endophthalmitis, as in this case. The aggressive nature of enterococcal endophthalmitis and the potential for synergism with combination therapy may supersede concerns about the potential for aminoglycoside-related retinal toxicity. We selected intravitreal amikacin as it has a somewhat better toxicity profile than intravitreal gentamicin [7]. Regarding systemic therapy, ocular VRE infection has been successfully managed with agents such as intravenous linezolid [2,5] and penicillin G [3]. The single report of quinupristin/dalfopristine use in an immunocompromised patient with VRE endophthalmitis resulted in panuveitis and enucleation [1]. Intravenous daptomycin may be another potentially useful agent, with known efficacy against VRE and good intravitreal penetration. In this case, prompt treatment in accordance with organism culture and sensitivities afforded a favorable outcome for this patient.

## Consent

Written informed consent was obtained from the patient for publication of this report.

## Abbreviations

IOFB: intraocular foreign body; VRE: vancomycin-resistant enterococcus

## Competing interests

The authors declare that they have no competing interests.

## Authors' contributions

RJH managed the case and drafted the final manuscript. PA performed a literature review and drafted the initial manuscript. GR managed the case and revised the manuscript critically for important intellectual content (from an infectious disease perspective). MO supervised the management of the case and revised the manuscript critically for important intellectual content (from an infectious disease perspective). RHM supervised the management of the case and revised the manuscript critically for important intellectual content (from an ophthalmic perspective). All authors read and approved the final manuscript.
